# Human induced pluripotent stem cell-derived neural stem cells survive, migrate, differentiate, and improve neurologic function in a rat model of middle cerebral artery occlusion

**DOI:** 10.1186/scrt224

**Published:** 2013-06-14

**Authors:** Ting Yuan, Wei Liao, Nian-Hua Feng, Yuan-Lei Lou, Xin Niu, Ai-Jun Zhang, Yang Wang, Zhi-Feng Deng

**Affiliations:** 1 Department of Orthopaedic Surgery, Shanghai Jiaotong University Affiliated Sixth People’s Hospital, Shanghai 200233, China; 2 Department of Neurosurgery, Shanghai Jiaotong University Affiliated Sixth People’s Hospital, Shanghai 200233, China; 3 Department of Neurosurgery, The Second Affiliated Hospital of Nanchang University, Nanchang, 330006, China; 4 Institute of Urology, The First Affiliated Hospital of Nanchang University, Nanchang 330006, China

**Keywords:** Induced pluripotent stem cell, Stoke, Neural stem cell, Middle cerebral artery occlusion

## Abstract

**Introduction:**

Stroke is a major cause of permanent neurologic damage, with few effective treatments available to restore lost function. Induced pluripotent stem cells (iPSCs) have the potential to generate all cell types *in vitro* and can be generated from a stroke patient. Therefore, iPSCs are attractive donor sources of genetically identical “patient-specific” cells to hold promise in therapy for stroke. In the present study, we established a four-stage culture system by using serum-free medium and retinoic acid (RA) to differentiate iPSCs into neural stem cells (NSCs) effectively and stably. Our hypothesis was that iPSC-derived NSCs would survive, migrate, and differentiate *in vivo*, and improve neurologic function after transplantation into the brains of rats with ischemic stroke.

**Methods:**

Human iPSCs (iPS-S-01) and human ESCs (HuES17) were used to differentiate into NSCs by using our four-stage culture system. iPSCs and differentiated NSCs were characterized by immunocytochemistry staining and reverse transcription-polymerase chain reaction (RT-PCR) analysis. After establishment of focal cerebral ischemia with occlusion of the middle cerebral artery (MCA) and cell transplantation, animals were killed at 1 week and 2 weeks to analyze survival, migration, and differentiation of implanted cells in brain tissue. Animal behavior was evaluated via rope grabbing, beam walking, and Morris water maze tests.

**Results:**

iPSCs were efficiently induced into NSCs by using a newly established four-stage induction system *in vitro*. iPSCs expressed pluripotency-associated genes *Oct4*, *Sox2*, and *Nanog* before NSC differentiation. The iPSC-derived NSCs spontaneously differentiated into neurons and astrocytes, which highly express β-tubulin and glial fibrillary acidic protein (GFAP), respectively. On transplantation into the striatum, CM-DiI labeled iPSC-derived NSCs were found to migrate into the ischemia area at 1 week and 2 weeks, and animal-function recovery was significantly improved in comparison with control groups at 3 weeks.

**Conclusions:**

The four-stage induction system is stable and effective to culture, differentiate, and induce iPSCs to NSCs by using serum-free medium combined with retinoic acid (RA). Implanted iPSC-derived NSCs were able to survive, migrate into the ischemic brain area to differentiate into mature neural cells, and seem to have potential to restore lost neurologic function from damage due to stroke in a rat model.

## Introduction

Stroke is one of the leading causes of death and disability in humans. Currently, no effective treatments can restore lost neurologic function after stroke in the clinic [1,2]. An increasing number of studies have provided evidence that cell transplantation can lead to the repair of damaged brain tissue and functional recovery in preclinical stroke models [3-8], including transplantation of bone marrow stromal cells (BMSCs) [9,10], neural stem cells (NSCs) [11,12], and embryonic stem cells (ESCs) [13,14]. These stem cells can differentiate into neural lineages to replace lost neurons and partially reconstruct damaged neuronal circuitry. Nevertheless, disadvantages such as immune rejection and less differentiation potential of adult stem cells, as well as ethical considerations of ESCs, now limit their utility in clinical trials.

The establishment of induced pluripotent stem cells (iPSCs) offers new prospects for regenerative medicine in stroke. iPSCs behave in a manner similar to that of ESCs with high reproduction ability and pluripotency to differentiate into various types of cells [15]. Furthermore, iPSCs can be generated from cells of any part of an adult patient, avoiding both ethical problems and immune rejection, and have a potential to provide genetically identical “patient-specific” cells for stroke patients.

iPSCs have been shown to differentiate successfully into neural precursor cells, neurons, hematopoietic cells, cardiomyocytes, smooth muscle cells, vascular endothelial cells, and so on, *in vitro*[16-19]. Some *in vivo* studies have confirmed that iPSCs transplanted directly into the cerebral tissue of ischemic stroke rats could differentiate into neurons and promote functional recovery [20,21], but only a very small number of implanted cells exhibited the specific markers of neurons. Furthermore, a portion of grafted iPSCs formed tumors in implanted sites [21]. These results indicate that the differentiation ability of iPSCs into neurons is limited and associated with unacceptable high rates of tumor formation. Therefore, if iPSCs are induced into NSCs before transplantation *in vitro*, it is possible to improve the effectiveness of cell therapy, while reducing the risk of tumor formation.

Retinoic acid (RA) has been shown to have the potential to improve the differentiation of ESCs into NSCs [22,23], but the efficiency is low. In recent years, the alternative serum-free induction protocols have been used widely to differentiate ESCs to NSCs for the advantage of definite components of culture medium, but this induction process needs a long time [24,25]. We used a modified protocol (a four-stage culture system) combining RA induction and serum-free medium culture. The human iPSCs and ESCs were efficiently induced into neural precursor cells and then differentiated into mature neural cells in this newly established four-stage culture system. After we induced them from iPSCs to NSCs, we implanted iPSC-derived NSCs stereotactically into the striatum of rats with ischemic stroke. To evaluate their therapeutic potential, we observed the differentiation, migration, and distribution of grafted cells after transplantation, and analyzed the neurologic and functional status. To our knowledge, we are the first to study the effects of combining RA and serum-free medium to induce iPSCs into NSCs and observe the direct migration of human iPSC-derived NSCs from implanted sites to ischemic brain areas to restore lost neurologic function in a rat model.

## Materials and methods

### Human iPSCs culture

Human iPSCs (iPS-S-01) and human ESCs (HuES17) were provided by the Institute of Biochemistry and Cell Biology of the Chinese Academy of Sciences in agreement with Liao J and Xiao L [26]. The iPS-S-01 cells were induced by six transcription factors (*Oct4*, *Sox2*, *Nanog*, *Lin28*, *C-myc,* and *Klf4*) and resembled the phenotype and pluripotency of undifferentiated human ESCs [26]. Both iPSCs and ESCs were maintained on mouse embryonic fibroblast (MEF) feeder layer, which was inactivated by mitomycin. The medium was changed every day. In general, every 5 to 7 days, when colonies approached approximately 70% confluence, or greater than 700 microns, the cultured cells were then passaged by 1 mg/ml collagenase type IV and mechanical dissociation, and split at a 1:4 to 1:6 ratio. The medium for iPSCs and ESCs culture consisted of Knockout DMEM supplemented with 20% Knockout serum replacement, 1% nonessential amino acid, 1 m*M* L-glutamine, 0.1 m*M* β-mercaptoethanol, and 4 ng/ml bFGF (Gibco, Grand Island, NY, USA).

### Neural differentiation

**Figure 1:**
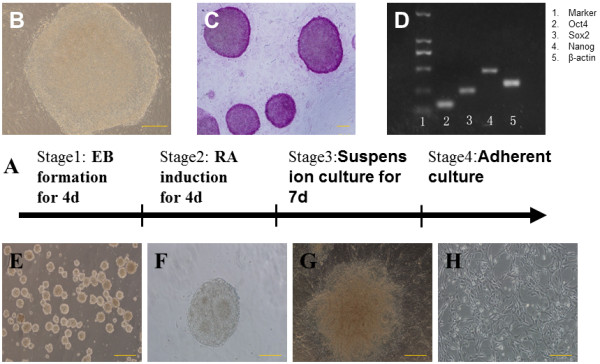
**Cell culture and induction of NSCs from iPSCs (bar = 50 μm).** The stages for neuronal differentiation **(A)**. iPSCs culture and identification (**B** through **H**). iPSCs maintained on a feeder layer tended to form packed clones with a high nucleus-to-cytoplasm ratio **(B)**, displayed a high level of alkaline phosphatase **(C)**, RT-PCR shows that iPSCs expressed pluripotent genes *Oct4*, *Sox2*, *Nanog***(D)**. EBs in suspending condition for 4 days and initiated the differentiation program **(E)**, spheres formed neural-tube like rosette structures **(F)**, neural-tube-like rosette structures in the center of adherent spheres **(G)**, network-like structure formed after the spheres were cultured in a laminin-coated flask for 1 month **(H)**.

Our protocol for neural differentiation is outlined later (Figure 1A) and divided into four stages. Colonies of iPSCs and ESCs were detached and pooled together in EB (embryoid body) medium (iPSCs medium without bFGF) in suspension culture in low-attachment Petri dishes for 4 days and grown as EBs (stage 1), which further were used for neural differentiation. The collected EBs were divided into three groups randomly: spontaneous differentiation group (EBs were cultured in EB medium for 11 days), retinoic acid (RA) induction group (EBs were in adherent culture in EB medium supplied with 5 × 10^-7^ *M* RA for 4 days, and then transferred to EB medium for 7 days), RA and serum-free medium induction group (EBs were cultured in EB medium supplied with 5 × 10^-7^ *M* RA for 4 days and then transferred to serum-free medium for 7 days) (stage 2). The serum-free medium consisted of DMEM/F12 (1:1), EGF (20 ng/ml), bFGF (10 ng/ml), B27 (2%), LIF (10 ng/ml), and heparin sodium (2 μg/ml) (all from Gibco). When RA-induced aggregates grew in media without RA, some cells detached from dish bottom and suspended and grew (stage 3). These suspended cells then formed neural spheres, which were then plated on poly-1-ornithine (10 mg/ml)/laminin (5 μg/ml)-coated dishes in the serum-free medium for adherent culture up to 7 days (stage 4). The cells were split 1:3 to 1:4 with a cell-detachment enzyme (Accutase) every 5 to 7 days.

### Establishment of focal cerebral ischemia and cell transplantation

All animal procedures were approved by the Institutional Animal Care and Use Committee of Jiangxi Province, China. All experiments on animals were carried out in an ethical manner. Adult male Sprague–Dawley rats (250 to 300 g) were used to establish the model of middle cerebral artery occlusion (MCAO), as previously described [27]. Rats were anesthetized, and a 4–0 nylon intraluminal suture was introduced from the minimal incision of the external carotid artery into the internal carotid artery at about a depth of 18 mm to 20 mm until it blocked the origin of the MCA. After 2-hour temporary MCA occlusion, reperfusion was performed by withdrawal of the suture. The rats with significant neurologic deficit of the left forelimb, according to the Longa 5-point scale [27], were chosen as candidates for cell transplantation. Sham-operated animals underwent the same surgical procedure without suture insertion.

**Figure 2:**
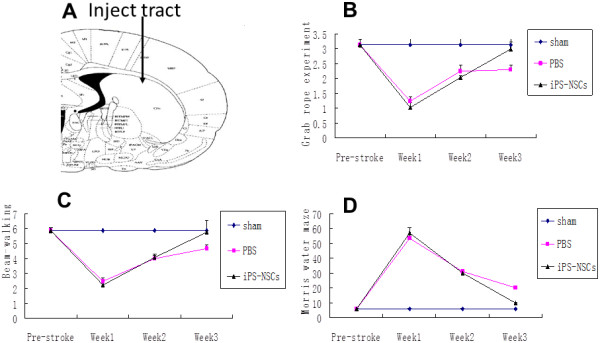
**Transplanted cells improved the behavioral and sensorimotor function of the models.** The NSCs derived from iPSCs cultured in RA and serum-free medium for 1 month were transplanted into the striatum of the animal brains **(A)**; rope-grabbing experiment **(B)**, beam-walking **(C)**, and Morris water maze **(D)** score. (Compared with the sham and PBS groups).

Two days before cell transplantation, all rats were immunosuppressed with a daily injection of cyclosporin A (20 mg/kg) and lasting for 1 week [28]. The NSCs were dissociated with Accutase to single cells, then were collected and labeled with CM-DiI before injection into the ischemia striatum (Figure 2A) by using a stereotactic apparatus (Zhenghua Biological Instruments Co., Ltd. Anhui Province, China). Based on a previously published article [29], the iPSC-derived NSCs (10^6^ cells in 30 μl PBS) solution was injected into the striatum site, which was 0.8 mm behind the anterior fontanel, 3.4 mm left to the sagittal suture, and the injection depth was 5.8 mm. The injection was performed immediately once the reperfusion began, the time was controlled within 10 minutes for a constant speed, and the needle was left in for an additional 5 minutes before removal. The rats were randomly divided into three groups (each group, *n* = 5): sham group, iPSCs-derived NSCs transplantation group, PBS transplantation group; and the transplantation group was further divided into two subgroups: transplantation for 7 days, transplantation for 14 days. The neurologic scores were determined before and after the cell transplantation. After 7 and 14 days from cell transplant, the rats were killed after deep anesthesia and were transcardially perfused with 4% paraformaldehyde. Dissected whole brains were fixed with 4% paraformaldehyde for 2 hours and were dehydrated by using sucrose density gradient at 20% to 30% for each day. The 8-μm frozen sections were prepared for immunocytochemistry staining.

### Immunocytochemistry staining

Cells and rat brains were fixed with 4% paraformaldehyde for 15 minutes. Nonspecific sites were blocked with 1% BSA/PBS (for extracellular epitopes) or 1% BSA/PBS/0.5% triton X-100 (for intracellular epitopes) for 30 minutes at 37°C. Then the specimens were incubated with primary antibodies at 4°C overnight. The redundant primary antibodies were removed by washing with PBS for 5 minutes and repeated 3 times the next day. Second antibodies labeled with FITC were used. Nuclei were stained with 4,6-diamidino-2-phenylindole (DAPI, 0.5 μg/ml, Invitrogen, Grand Island, NY, USA). The following antibodies were used: Anti-*Nestin* (monoclonal, Santa Cruz Biotechnology, Santa Cruz, CA, USA), Anti-*Sox2* (monoclonal, Cell Signaling, Danvers, MA, USA), Anti-β-Tubulin (monoclonal, Chemicon, Billerica, MA, USA), Anti-glial fibrillary acidic protein (GFAP, monoclonal, Santa Cruz Biotechnology), Anti-*Oct3/4* (monoclonal, Cell Signaling, Danvers, MA, USA). Primary and secondary antibodies were diluted in 1% BSA (Roche, Indianapolis, IN, USA) at 1:100 and 1:200, respectively. Fluorescence was observed under a fluorescence microscope (Olympus, Tokyo, Japan), and photos were taken with a DP70 digital camera system (Olympus). Positive cells in brain tissue were counted blindly in five coronal sections per animal.

### Reverse transcription-polymerase chain reaction analysis

Total RNA was extracted from cultured cells by using Trizol reagent (Invitrogen). 1 μg of total RNA from the cells was used for the synthesis of the first-strand cDNA with a Revert Aid first-strand cDNA synthesis kit (Fermentas, Life Sciences, Burlington, ON, Canada) in a total volume of 20 μl. For quantitative PCR, SYBR Green supermix was used, and standard curves for each primer set were generated to confirm that only one amplicon was generated at the same efficiency as the reference gene GAPDH. PCR was performed by using the following thermal profile: 10 minutes at 95°C; denature at 95°C for 15 seconds, annealing for 30 seconds at 60°C, elongation at 72°C for 30 seconds, for 40 cycles; extension for 10 minutes at 72°C, and finally paused at 4°C. Relative expression was calculated by using the comparative Ct method (2^-△△Ct^). 2^-△△Ct^ >3 or <1/3 was deemed statistically significant. The PCR primers special to each transcript were as follows (Table 1). (All primers were designed by using the Primer Designer software).

**Table 1:** Primers used for reverse transcription-polymerase chain reaction (RT-PCR) analysis

**Genes**	**Forward (5’–3’)**	**Reverse (5’–3’)**
***GAPDH***	GGCATGGACTGTGGTCATGAG	TGCACCACCAACTGCTTAGC
***Oct4***	CGA AGAGAAAGCGAACCA GTATC	AGAACCACACTCGGACCACATC
***Sox2***	CCCC CGG CGGCAATAGCA	TCGGCGCCGGGG AGATACAT
***Nestin***	GCCCTGACCACTCCAGTTTAG	CCCTCTATGGCTGTTTCTTTCTCTAC
***GFAP***	GAAGCTCCAGGATGAAACCA	ACCTCCTCCTCGTGGATCTT
***β-tubulin***	CTGGTGGAGAACACGGATGAG	GCGGAAGCAGATGTCGTAGAG

### Neurobehavioral evaluation

Neurobehavioral evaluation was performed according to the previously published studies [30-32]. The rats were subjected to behavioral testing 1, 2, and 3 weeks after MCAO modeling, by using the rope-grabbing experiment for assaying the myodynamics of the model, beam walking and Morris water maze for testing the spatial, working memory of the models.

### Statistical analysis

Outcome measurement for each experiment was reported as mean ± SEM. All data were analyzed by using SPSS 17.0. Significance of intergroup differences and nerve-assessment data were analyzed with repeated measures of analysis of variance (ANOVA), and the unpaired Student *t* test. A *P* value of less than 0.05 was considered to be statistically significant.

## Results

### IPSCs efficiently induced into NSCs with RA and serum-free medium

iPS-S-01 and HuES17 tended to form packed clones with a high nucleus/cytoplasm ratio (Figure 1B) and displayed a high level of alkaline phosphatase (Figure 1C). To make sure of the undifferentiated state of the cells, pluripotency-associated genes *Oct4*, *Sox2,* and *Nanog* were detected with qRT-PCR before the NSC differentiation. The results showed that those cells expressed high levels of pluripotency-associated genes (Figure 1D).

**Figure 3:**
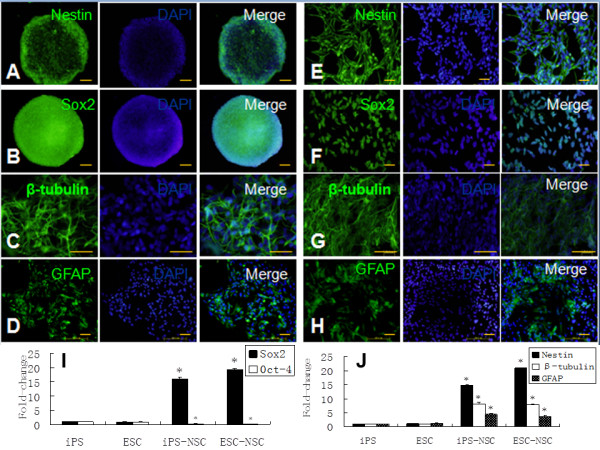
**The expression of pluripotent potent and neural markers during the induction process of the iPSCs to NSCs.** Immunocytochemistry staining shows that the spheres expressed the neural precursor markers *Nestin***(A)**, *Sox2***(B)** induced by RA and serum-free medium, and after culture, in serum medium; those spheres expressed the neuron marker β-tubulin **(C)** and astrocyte marker GFAP **(D)**. The adherent cells expressed the neural precursor markers *Nestin***(E)**, *Sox2***(F)**, and differentiated into neurons and astrocytes in the same conditions **(G, H)**. The expression of pluripotent genes *Sox2* and *Oct4* during the induction process in the iPSCs and ESCs **(I)**; the iPSC-derived NSCs expressed high levels of *Nestin*, β-tubulin, and GFAP during the induction process and showed a similar tendency to the ESC-derived NSCs (**J**, *compared with ESCs; *P* < 0.05).

A four-stage induction protocol was used for iPS-S-01 and HuES17 differentiation into the NSC. iPS-S-01 and HuES17 clones were cultured in suspension condition for 4 days and formed embryonic bodies (EB) (Figure 1E). Then these EBs were transferred to serum-free medium supplied with 5 × 10^-7^ *M* RA for another 4 days of induction, which promoted the neutralization program. Neural-tube-like rosette structures appeared on the third day (Figure 1F), with the more culture days, the more rosette structures. These spheres expressed uniformly for *Nestin* and *Sox2* (Figure 3A,B) with immunocytochemistry staining. When plated on poly-L-ornithine and laminin precoated flasks and cultured in serum-free medium, iPSC-derived NSCs could also form neural-tube-like rosette structures (Figure 1G). The network-like structures formed after the spheres were cultured in a laminin-coated flask for 1 month (Figure 1H). Accutase was used for the passage of those cells. Immunocytochemistry staining showed that those cells could express *Nestin* and *Sox2* as highly as before (Figure 3E,F). Whereas the iPSCs-derived NSCs were cultured in medium supplement with fetal bovine serum, iPSCs-derived NSCs spontaneously differentiated to neurons and astrocytes, which highly express β-tubulin and GFAP, respectively (Figure 3C,D,G,H). The qRT-PCR results demonstrated that the pluripotent stem cell gene *Sox2* expression was increased, and *Oct4* expression was decreased during the induction process (Figure 3I). Both the iPSC-derived NSCs and the ESC-derived NSCs expressed high levels of *Nestin*, β-tubulin, and GFAP (Figure 3J).

**Figure 4:**
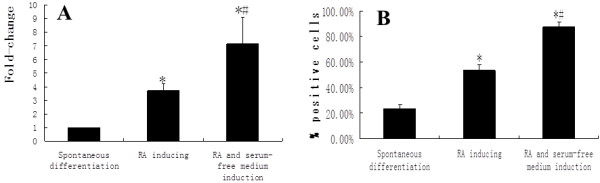
**The expression of *Nestin *during the induction process among the three groups.** The quantity of mRNA of *Nestin* in RA plus serum-free medium group was the highest among the three groups **(A)**. During the induction process, *Nestin*-positive cell rate was the highest in RA plus serum-free-medium group **(B)** (*compared with the spontaneous-differentiation group, *P* < 0.05; #compared with the RA-induction group, *P* < 0.05).

To test whether iPSCs cultured in serum-free medium combined with RA could be efficiently differentiated into NSCs, we chose three groups to compare: RA induction group, RA and serum-free medium induction group, and spontaneous differentiation group. The cells were collected on days 7. The qRT-PCR results indicated that the expression levels of *Nestin* in RA induction group as well as RA and serum-free medium induction group were significantly higher than the spontaneous differentiation group. The fold changes were 3.71 ± 0.63 and 7.14 ± 1.96, respectively (Figure 4A). Additionally, the immunostaining results showed that *Nestin*-positive cell rates were 23.77% ± 2.96%, 53.25% ± 4.52%, and 87.54% ± 3.67% (Figure 4B) in the spontaneous-differentiation group, RA-induction group, and RA plus serum-free medium-induction group, respectively. Significant differences were found between groups. It means that the iPSCs can be induced into NSCs highly efficiently with RA and serum-free medium.

### Transplanted cells survived and migrated into the stroke-damaged host tissue

**Figure 5:**
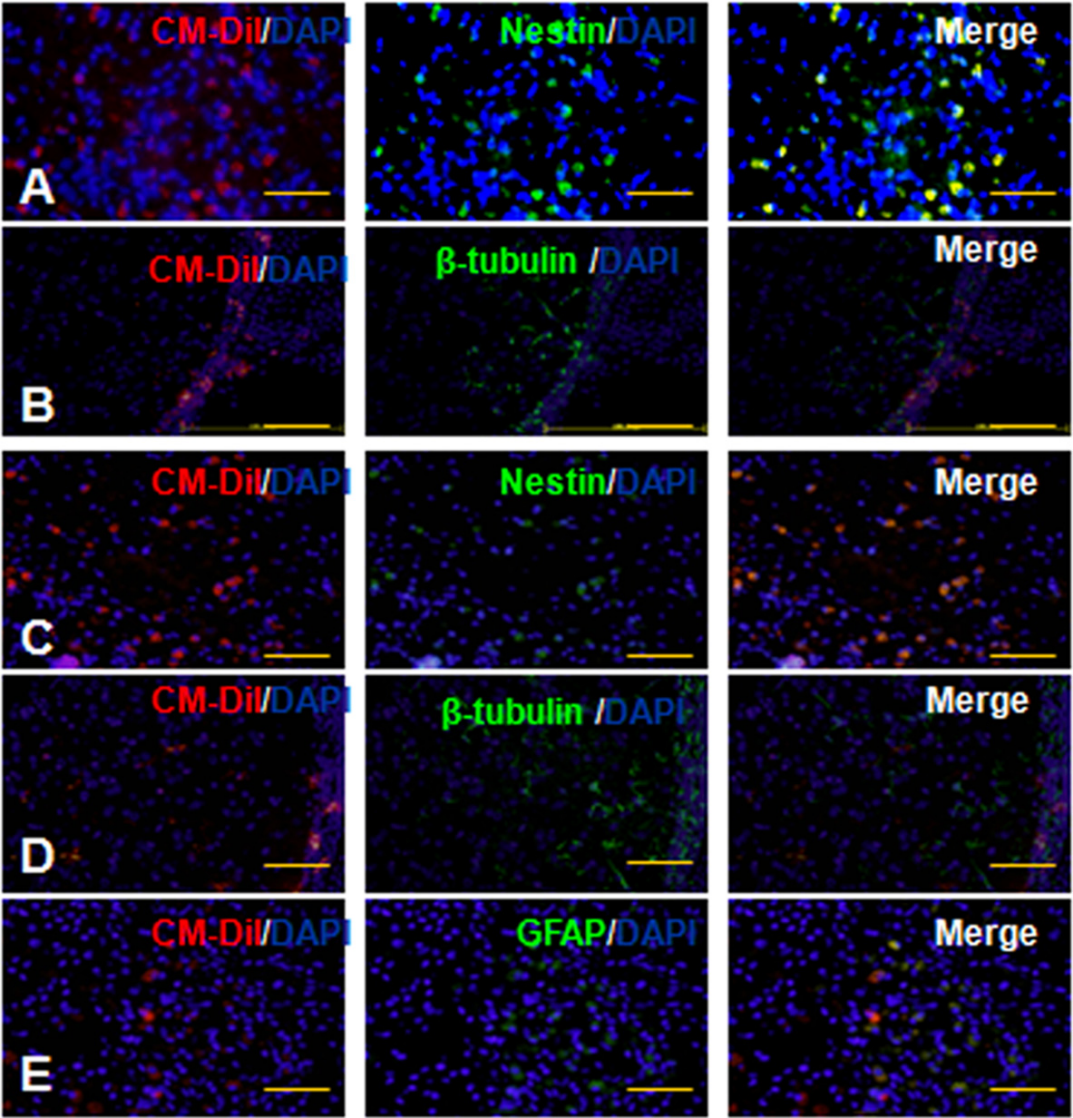
**Transplanted cells survived and migrated into the stroke-damaged host tissue (bar = 100 μm).** The NSCs derived from iPSCs differentiated into neural cells, which were immunopositive for *Nestin***(A, C)** and β-tubulin **(B, D)** (**A**, **B**: group of transplantation for 7 days; **C**, **D**: group of transplantation for 14 days). Two weeks after transplantation, some of the transplanted cells were immunopositive with GFAP **(E)**.

The brain tissues from weeks 1 and 2 processed for immunocytochemistry staining showed that: amounts of CM-DiI-labeled NSCs derived from iPSCs were found besides the striatum after the cells were transplanted. One week later, the CM-DiI-labeled cells (68.2 ± 2.3 count/field) were found in the models’ brains, particularly around the striatum, but less (11.6 ± 1.8 count/field) in the other areas, which demonstrated that the transplanted cells could survive in the models. Two weeks after transplantation, the majority of cells (37.2 ± 3.5 count/field) migrated into the ischemia area. The immunocytochemistry staining showed that the grafted cells expressed the *Nestin* (68.5% ± 2.8%, Figure 5A), and a few cells expressed the β-tubulin (25.7% ± 12.3%; Figure 5B) for the 1-week transplantation group. The 2-week transplantation group (51.4% ± 7.5% Figure 5C) expressed *Nestin*, and more grafted differentiated into the neuron (44.3% ± 2.5% expressed β-tubulin Figure 5D) and astrocytes (11.2% ± 1.4% expressed GFAP; Figure 5E). These results suggested that the NSCs derived from iPSCs could survive in the models of MCAO and differentiate into the neurons and astrocytes. We did not observe the tumorigenesis during the transplantation process.

### Transplanted cells improved the behavioral and sensorimotor function of the rats

Animal behavior was evaluated with the rope-grabbing, beam-walking, and Morris water-maze tests 0 days, 1, 2, and 3 weeks after MCAO models were prepared. No obvious differences among the groups were found before the MCAO or transplantation. It was found that no obvious sensorimotor and functional differences among the three groups at 0 days and the first and second weeks (*P* > 0.05); but at the third week, the transplantation group showed greater function recovery than did those of the other groups (PBS and sham groups, *P* < 0.05) (Figure 2B through D). These results demonstrate that the NSCs derived from iPSCs improved the behavioral and sensorimotor functions in a rat model of MCAO through transplanting cells in the striatum.

## Discussion

In recent years, with the development of regenerative medicine, stem cell transplantation for the treatment of central nervous system disease is considered to be a way of great potential. NSCs derived from iPSCs seem to be the ideal seed cells for transplantation therapy for neurologic diseases. Because iPSCs share similar characteristics and potential with ESCs, we may study iPSCs by applying the same protocols that had been previously developed in ES cells.

Various culture protocols for the generation of NSCs from iPSCs have been reported in many studies, and the main methods are the following: RA-mediated induction [33], stromal cell coculture, conditioned medium induction, and serum-free-medium induction [25]. RA has been demonstrated to induce significant neural differentiation of ESCs [33-35]. In 1995, Bain [34] used RA to induce mouse ESCs into nerve cells successfully, and these cells had the electrophysiological properties and functions of nerve cells. Later, Zhou and Bahrvand [36,37] reported that the use of RA could promote human ESCs differentiation into neural stem cells, but efficiency was low, and the survival capacity of RA-induced neuronal cells was limited [38]. Stromal cell coculture and conditioned-medium induction are also able to induce ESCs to differentiate into neural cells, but the two methods have a common shortcoming, in that the medium composition is complex, and this complicates the study of its mechanism.

In recent years, induction protocols using cytokines and serum-free medium have been established. Koch [24] successfully used serum-free medium to induce human ESCs into NSCs. The serum-free medium contains N2, insulin, FGF, and B27, and the data showed that FGF was able to stimulate proliferation and differentiation of a variety of cell types originated from multigerminal layers. A study [39] showed that neuroepithelial precursor cells differentiated from ESCs proliferate in the presence of basic fibroblast growth factor (bFGF), and differentiate into both neurons and glia after withdrawal of bFGF. Daadi [25] successfully induced ESCs into NSCs by using serum-free medium containing bFGF, leukemia inhibitory growth factor (LIF), and EGF. These factors are known to stimulate the proliferation of NSCs, and LIF has the potential to maintain the pluripotency of NSCs and to prevent NSCs from differentiating into neurons. Definite components of serum-free medium are beneficial to study the relation between cell growth and medium components.

In the present study, we established a modified four-stage culture system to induce iPSCs to NSCs by taking the advantages of previously developed protocols, by using RA combined with serum-free medium containing bFGF, EGF, LIF, B27, and heparin sodium to induce iPSCs to NSCs in adherent culture. The results showed that this system was stable and effective to induce neural differentiation of iPSCs. Induced cells expressed high levels of NSC markers, *Nestin* and *Sox2*, and have been amplified and passaged *in vitro* for more than 35 cell generations.

In addition, NSCs can be expanded and differentiated in both suspension and adherent cultures. However, it is difficult to control the quality and quantity of cells in the process of suspension culture, because ESC-derived neurosphere cells will lose the potential of self-renewal and differentiation gradually during the long suspension culture, and the types of neurosphere cells are very diverse [40-42]. The adherent culture may avoid these shortcomings.

After implantation into the brain in a rat model of stroke, iPSC-derived NSCs were able to survive and express NSC makers. One week after implantation, implanted cells expressed mainly β-tubulin in addition to *Nestin*; after 2 weeks, besides continuing to express *Nestin* and β-tubulin, a few implanted cells expressed GFAP (maker of astrocytes). These results show that grafted NSCs have the potential to differentiate into neurons and glia, and create the possibility of repairing brain injury or other neurologic defects through a neural cell-replacement strategy.

Tumorigenesis is a big challenge in stem cell-replacement therapy. Some animal studies demonstrated that direct implantation of ESCs or iPSCs led to tumor formation [21,43]. In this study, two rats survived for more than 3 months, and no abnormal proliferation of cells was observed (data not shown). It implied that the implanted cells were not susceptible to tumorigenesis. Although we did not observe tumor formation in this study, it could be that a short period of observation lasting for only 2 weeks was not long enough for tumor development and formation. Therefore, the long-term safety of iPSC-derived NSCs implantation is still a serious problem.

Grafted stem cells have the potential to migrate *in vivo*. A number of studies [44,45] observed migration of various implanted stem cells into lesions of the brain, and indicated that possibly stem cells are targeted by inflammatory chemotactic factors and cytokines extracted from ischemic brain tissue. This feature of chemotaxis is the pathophysiologic basis of repairing brain injury by cell-implantation therapy. In this study, implanted NSCs were observed migrating from the striatum toward the ischemic boundary at 2 weeks after implantation.

In our study, we observed a significant increase of neurologic scores 3 weeks after transplantation compared with the control groups, demonstrating a positive effect of repairing ischemic brain with iPSC-derived NSCs. Although 3 weeks is long enough for differentiation of implanted NSCs into neurons, it is not enough for implanted cells to replace dead neurons and function. As a result, some scholars [46,47] believe that early recovery of function with stem cell transplantation is due to neurologic protection of growth factor release, not new neural cells replacement. Therefore, restoration of neurologic function in this study after cell implantation may be more relevant to a neuroprotective effect.

Note that PBS injection also improved the behavior of rats at 2 weeks (Figure 2B through D). One reason is that collateral circulation has been formed in brain tissue, and the function of impaired cells may be recovered in 2 weeks, which contributes to the improved behavior of rats. Moreover, the improvement of behavior in this rat model may be partly owing to functional compensation after brain injury.

## Conclusions

This study established a stable and effective culture system for cell expansion, differentiation, and induction of iPSCs to NSCs *in vitro* by using serum-free medium combined with RA. After stereotactic implantation of iPSC-derived NSCs into the brain in a rat-stroke model, implanted cells were able to survive, migrate into ischemic brain areas, and differentiate into mature neural cells. Furthermore, this cell-implantation therapy seemed to restore lost neurologic function to a certain extent in a rat stroke model.

## Abbreviations

bFGF: Basic fibroblast growth factor; BMSC: Bone marrow stromal cell; BSA: Bovine serum albumin; DAPI: 4, 6-Diamidino-2-phenylindole; DMEM: Dulbecco modified Eagle medium; EB: Embryoid body; EGF: Epidermal growth factor; ESC: Embryonic stem cell; FITC: Fluorescein isothiocyanate; GFAP: Glial fibrillary acidic protein; iPSC: Induced pluripotent stem cell; LIF: Leukemia inhibitory factor; MCAO: Middle cerebral artery occlusion; MEF: Mouse embryonic fibroblast; NSC: Neural stem cell; PBS: Phosphate-buffered saline; RA: Retinoic acid; RT-PCR: Reverse transcription-polymerase chain reaction

## Competing interests

The authors declare that they have no competing interests.

## Authors’ contributions

ZFD and YW conceived the idea and designed the experiments, TY and WL performed animal experiments, data analysis, and manuscript preparation. NHF, YLL, and XN are responsible for cell-culture experiments and the collection and assembly of data. AJZ contributed to animal experiments and neurologic-function analysis. All authors read and approved the final manuscript.
